# Retrograde Arterial Flow Secondary to Median Arcuate Ligament Syndrome as a Contraindication to Gastroduodenal Artery Angioembolization

**DOI:** 10.7759/cureus.67130

**Published:** 2024-08-18

**Authors:** Joey Almaguer, Sheedeh Motamedi, Dylan Murray, Matthew Murray, Richard Murray

**Affiliations:** 1 Radiology, Texas Tech University Health Sciences Center, Amarillo, USA; 2 Radiology, Texas Tech University Health Sciences Center, Lubbock, USA; 3 Surgery, University College Dublin, Dublin, IRL; 4 Surgery, Royal College of Surgeons, Dublin, IRL; 5 Diagnostic and Interventional Radiology, Northwest Texas Healthcare System, Amarillo, USA

**Keywords:** angioembolization, contraindication, gastroduodenal artery, retrograde flow, gi bleed, duodenal ulcer, peptic ulcer disease, mals, median arcuate ligament syndrome

## Abstract

Median arcuate ligament syndrome (MALS) is a rare condition in which the median arcuate ligament (MAL) exerts external compression on the celiac trunk. Most cases are asymptomatic and diagnosed incidentally on radiographic imaging; however, some patients may experience gastrointestinal (GI) symptoms related to foregut ischemia and/or celiac neuropathy. In the following case, we present a patient with hemorrhagic peptic ulcer disease of the duodenum, which resulted in episodes of hemodynamic instability requiring multiple blood transfusions. Upon attempted transarterial angioembolization of the gastroduodenal artery (GDA), celiac stenosis and retrograde arterial flow from the superior mesenteric artery confirmed the presence of MALS. This rendered GDA angioembolization a contraindication, as the GDA became the dominant arterial supply for the distal celiac organs. The patient then received open surgical MAL release with concurrent surgical ligation of the hemorrhaging duodenal artery, which resolved his symptoms without the need for further intervention.

## Introduction

Median arcuate ligament syndrome (MALS), also known as celiac artery compression syndrome or Dunbar syndrome, is a rare condition in which the median arcuate ligament (MAL) of the diaphragm compresses the celiac trunk. The celiac trunk normally branches off the abdominal aorta between T11-L1 vertebral levels, whereas the diaphragmatic crura normally arises between the L1-L4 vertebral levels [1]. The MAL connects the crura of the aortic hiatus and normally passes over the celiac trunk at the L1 vertebral level [2]. Individuals with a high-riding origin of the celiac trunk and/or a low-riding level at which the MAL crosses the abdominal aorta have a higher likelihood of developing MALS [3]. Classic radiographic findings of MALS include a hooked appearance (“J-hook” sign) at the site of compression with resultant post-stenotic dilation of the celiac artery [4].

The majority of patients with MALS are asymptomatic and only diagnosed incidentally on radiographic imaging [4]. The presence or absence of symptoms could be attributed to whether collateral vasculature is present to prevent foregut ischemia as well as whether the celiac ganglion and/or plexus are also compressed by the MAL [5]. The degree of celiac trunk compression by the MAL also varies temporally due to diaphragmatic elevation and depression during respiration: inspiration relieves the compression on the celiac trunk, whereas expiration will exacerbate the compression. This is due to the joint caudal and ventral motion of the MAL during inspiration, which temporarily relieves its compression on the celiac trunk, allowing for transient anterograde blood flow [6].

If severe enough, the compression of the celiac trunk can manifest as diminished perfusion to distal celiac organ systems and/or celiac ganglion/plexus compression [7]. Resultant foregut ischemia and/or neuropathy can manifest symptomatically as resting, postprandial, or exercise-induced abdominal pain, decreased oral intake, unexplained weight loss, bloating, nausea, vomiting, or diarrhea [8]. On physical examination, an epigastric bruit on auscultation and/or abdominal tenderness on palpation may be present; however, these signs are nonspecific for MALS [9]. The abdominal pain can vary in severity, location, and duration, but tends to be epigastric and may be relieved by leaning forward or crouching [10].

The incidence of MALS is about two in every 100,000 patients and appears to be more common in women (4:1), those with a lower body mass index, and adults between 30 and 50 years old. [11-14]. Because of the relatively common symptom manifestation, MALS is typically considered a diagnosis of exclusion. Diagnosis of MALS can be made by duplex ultrasonography during respiration, gastric exercise tonometry, CT or magnetic resonance angiography (MRA) of the abdomen, mesenteric arteriogram, or pressure gradient measurements of the celiac artery [15]. Upon positive diagnostic findings for MALS, it is then necessary to determine if patient symptoms are present and whether the severity of these symptoms warrants therapeutic intervention.

The primary treatment for MALS is typically open, laparoscopic, or robotic MAL release [16,17]. If decompression alone does not result in optimal celiac arterial flow, concurrent graduated celiac dilation or celiac reconstruction can also be performed to combat the hyperplastic intimal changes associated with chronic compression [18]. Celiac ganglion/plexus excision or neurolysis may also be performed concurrently, depending on the presence and quality of neuropathic symptoms [19]. If symptoms persist after these interventions, transarterial angioplasty with placement of a balloon-expandable stent within the celiac trunk can be considered. Because angioplasty with stent placement alone does not address the underlying celiac trunk compression, it should only be performed after MAL release due to poor patient outcomes observed when performed as the first-line therapy [20]. If these interventions are unsuccessful at relieving the symptoms, celiac artery reconstruction with mesenteric bypass surgery or a diagnosis of functional gastrointestinal (GI) disorder can be considered [15, 21].

The following presented case involves a patient with chronic non-steroidal anti-inflammatory drug (NSAID) use and *Helicobacter pylori* (*H. pylori*) infection, resulting in severe peptic ulcer disease of the duodenum. The large, hemorrhagic duodenal ulcer caused multiple episodes of GI bleeding that ultimately resulted in hemorrhagic shock. Attempted gastroduodenal artery (GDA) angioembolization revealed retrograde flow of arterial blood originating from the superior mesenteric artery (SMA) via the GDA, rendering GDA angioembolization a contraindication. The patient then underwent open MAL decompression surgery and ligation of the bleeding artery to restore arterial flow through the celiac trunk and achieve hemostasis of the actively hemorrhaging duodenal ulcer, respectfully. The patient recovered well and has not returned for further treatment.

## Case presentation

A 39-year-old male without any significant medical history presented to the emergency department complaining of hematemesis of bright red blood, melena, and excessive nausea and vomiting. He had generalized abdominal, chest, and back pain for the past three months, which he had tried to mitigate with daily over-the-counter naproxen, ibuprofen, and AlkaSeltzer. He admitted to ingesting a box of ibuprofen each week in an attempt to reduce the severity of the pain. He was not taking any prescription medications at the time but smoked cigarettes (one pack per day for the last 20 years) as well as marijuana (14 g/day since the age of 18). He also had significant weight loss from lack of oral intake as a result of acid reflux, abdominal pain, and recurrent vomiting.

He was tachycardic at 123 beats per minute and hypotensive at 79/52 mmHg. Blood work revealed a hemoglobin level of 6.8 mg/dL and a hematocrit level of 24%, likely due to acute blood loss. The hemorrhagic shock was treated with two units of packed red blood cells (PRBC), which drastically improved his vital signs. There was no evidence of infectious or traumatic processes. Differential diagnoses included Mallory-Weiss tear, Boerhaave syndrome, and hemorrhaging peptic ulcer disease. There was a low suspicion for bleeding esophageal varices, as there was a lack of alcohol abuse, hepatitis, or any known liver disease.

**Figure 1:**
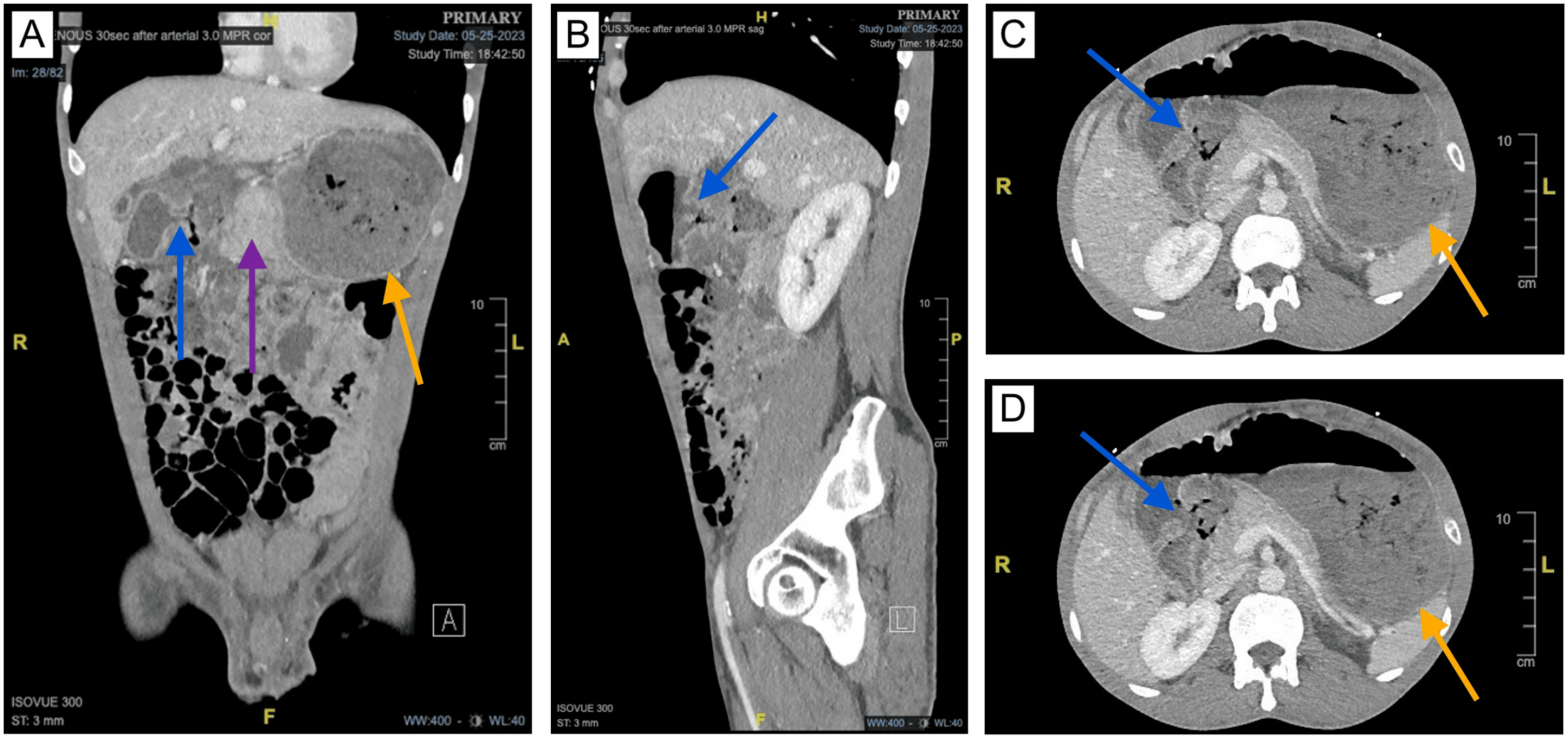
A CT without contrast demonstrates features of peptic ulcer disease of the duodenum. (A/B) Coronal and sagittal views show severe edema of the gastric pylorus and duodenum (purple arrow) as well as a possible contained perforation of the duodenal wall (blue arrows). (C/D) Axial views show the possible duodenal ulcer perforation that is walled off (blue arrows). The severe edema may be causing gastric outlet obstruction, which can be appreciated by the severely distended stomach with retained gastric contents (orange arrows).

A CT of the abdomen and pelvis with and without intravenous (IV) and oral contrast revealed severe edema of the gastric pylorus and the duodenum, which may be seen in peptic ulcer disease. There was also a small bowel intussusception in the right upper quadrant (RUQ) that was causing a moderate small bowel obstruction, likely involving the distal jejunal loops. There was swirling of the mesenteric ventricles within the central abdomen, suggesting an internal hernia. Lastly, there was increased soft tissue attenuation in the RUQ with apparent central necrosis, which could represent a large ulcer with surrounding gastritis or a perforated ulcer that is walled off (Figure 1). There was no evidence of active bleeding. The patient was then admitted into the medical intensive care unit for GI and general surgery consultation.

The patient was given another two units of PRBC. A nuclear medicine GI blood loss scan was ordered, which was negative for labeled red blood cell assessment for acute GI bleeding. The GI team then performed an esophagogastroduodenoscopy, which showed a large (3 cm) ulcer with a clot in the duodenal bulb without active bleeding. Pathologic evaluation of the gastric antral biopsy revealed chronic moderate gastritis with no evidence of gastric malignancy. The *H. pylori* immunohistochemistry was also performed, which returned positive. In relation to the small bowel intussusception resulting in a moderate small bowel obstruction, spontaneous resolution was determined to have occurred, as the patient had previously passed flatus and bowel movements and denied nausea, vomiting, and abdominal pain at the time of surgical evaluation.

**Figure 2:**
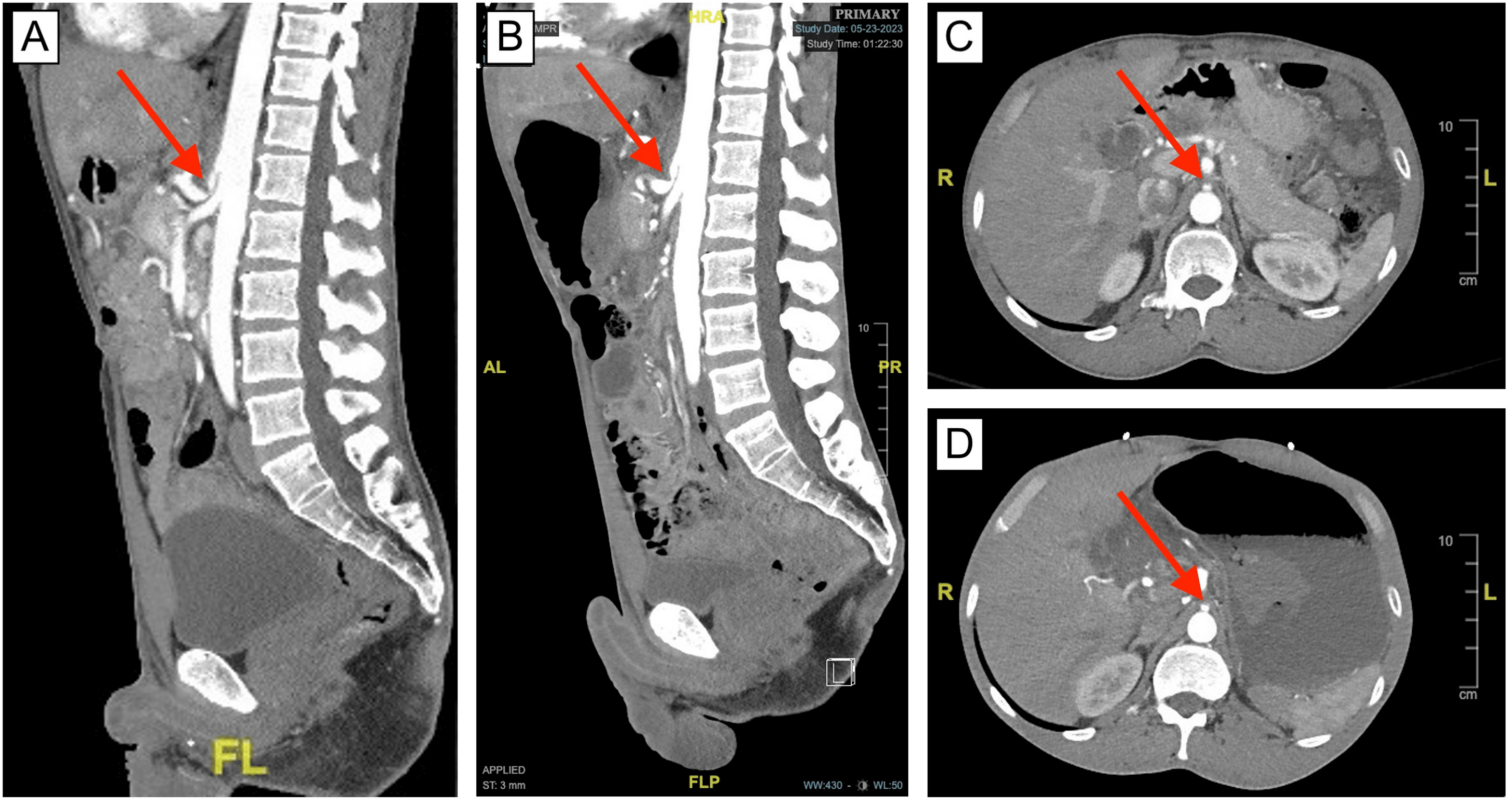
A CT angiography demonstrates compression of the celiac trunk in 2021 and 2023 (A/C) Sagittal and axial views of CT angiography taken in 2021 display the prototypical “J-hook” sign that is often associated with MALS and the location of the celiac trunk compression (red arrows). This medical encounter in 2021 was for a traumatic fracture of the right tibia and fibula, and the patient was not experiencing any GI symptoms at that time. (B/D) Sagittal and axial views of CT angiography taken in 2023 (time of relevant medical encounter) demonstrate celiac trunk compression (red arrows) imparted by the MAL. Post-stenotic dilation of the distal segment of the celiac artery can also be appreciated. There do not appear to be significant changes relative to the degree of celiac stenosis seen in 2021 (A/C) and 2023 (B/D).

The patient then suddenly began to experience active hematemesis and hematochezia and was treated with IV fluids, two additional units of PRBC, one unit of fresh frozen plasma (FFP), and tranexamic acid. He was then transferred to interventional radiology (IR) for GDA angioembolization. A review of the CT angiogram prior to the procedure suggested severe stenosis of the celiac trunk, likely due to MALS (Figure 2, frames B and D). The patient was placed under moderate IV sedation, and vascular access was achieved from the right common femoral artery under ultrasound guidance. Angiography from the abdominal aorta did not show any active bleeding from the celiac or superior mesenteric arteries.

**Figure 3:**
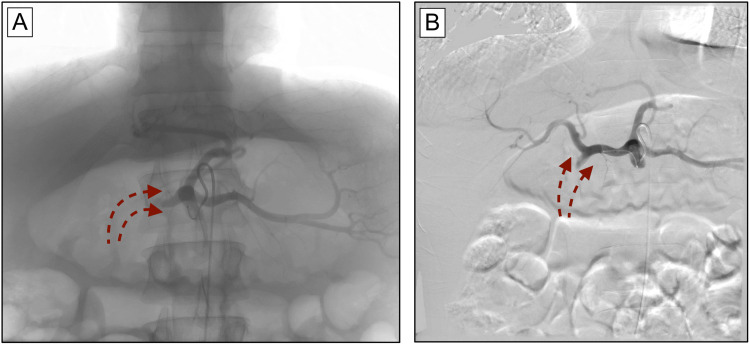
Fluoroscopic angiography reveals retrograde arterial flow originating from the GDA. (A/B) Lack of contrast enhancement of the GDA indicates retrograde arterial flow from the SMA through the pancreaticoduodenal arcades. Dotted maroon arrows demonstrate the direction of retrograde arterial flow. An incidental accessory left hepatic artery can also be seen branching off of the left gastric artery. GDA: gastroduodenal artery; SMA: superior mesenteric artery

**Figure 4:**
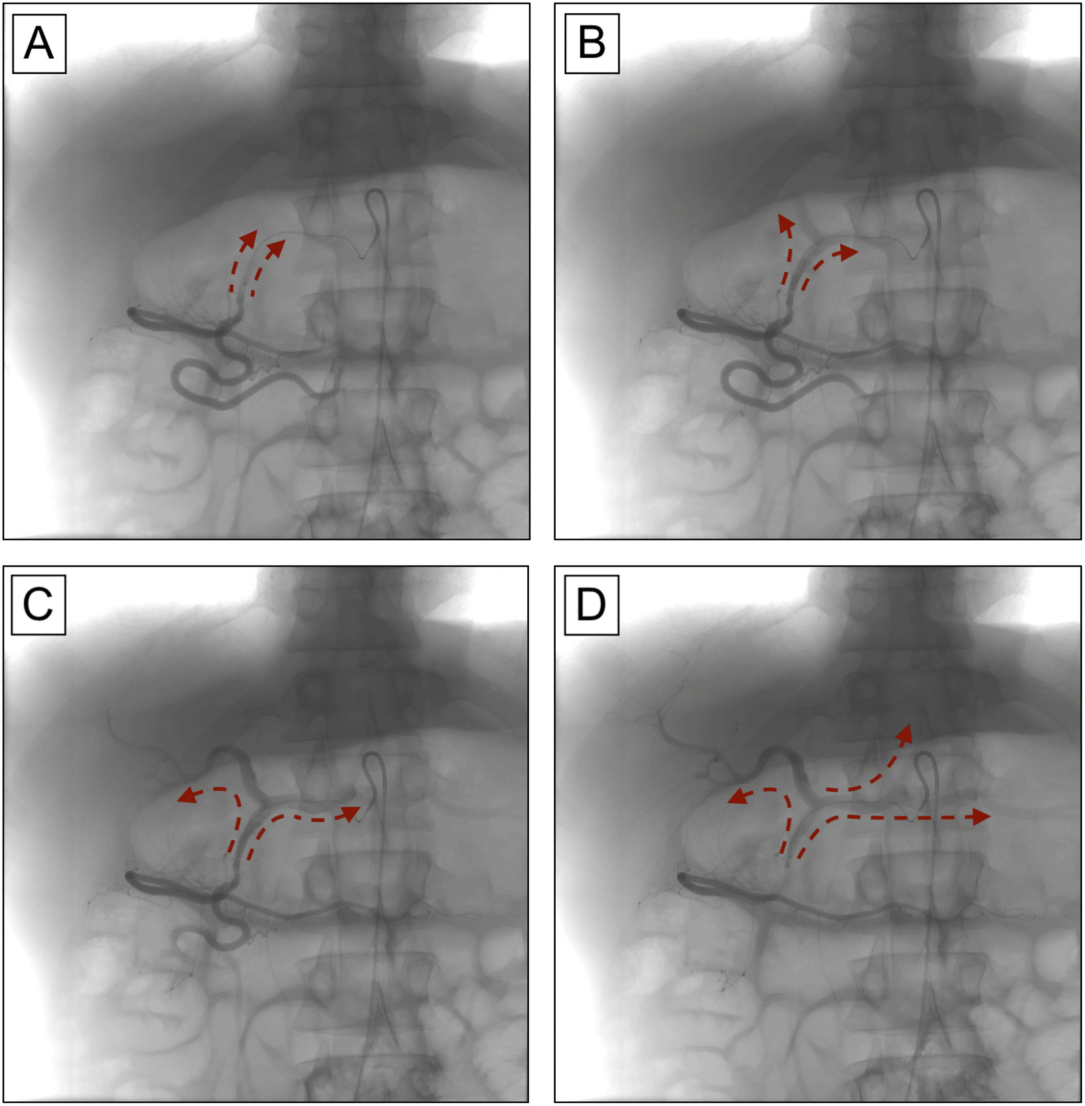
Fluoroscopic angiography at the level of the GDA demonstrates retrograde arterial flow. (A-D) The tip of the microcatheter can be seen at the distal portion of the GDA. Contrast delivery at this level over time reveals retrograde arterial flow from the GDA to the proper hepatic artery, left and right hepatic arteries, common hepatic artery, left gastric artery, and splenic artery. GDA: gastroduodenal artery

The celiac trunk was then accessed using an SOS Omni catheter. Injection of a small quantity of contrast showed stagnation of the contrast in the proximal centimeter of the celiac trunk, which was then dissipated upon the patient taking a deep breath. Next, a microcatheter was passed through the celiac stenosis, and angiography of the celiac trunk was performed through the microcatheter, which showed retrograde flow in the common hepatic artery. A microcatheter was placed in the common hepatic artery, and angiography showed retrograde flow from the GDA (Figure 3). The microcatheter was then advanced into the GDA, and subsequent angiography at this level did not reveal any active bleeding. However, continued retrograde flow from the SMA was observed (Figure 4).

The SOS Omni catheter was then placed into the SMA, and angiography at this level revealed retrograde flow through the pancreaticoduodenal arcades into the GDA as well as retrograde flow through the GDA into the common hepatic artery and the remainder of the celiac trunk, including the left gastric artery and splenic artery. These findings confirmed severe stenosis and functional occlusion of the celiac trunk due to MALS. There was a minimal amount of blood flow with both inspiration and expiration, likely due to the degree of compression of the celiac artery by the MAL. Given the angiographic findings of MALS, the GDA could not be prophylactically embolized, as this could result in further ischemia to the organs classically supplied by the celiac trunk, such as the liver, stomach, pancreas, and spleen.

As a chronic compensatory mechanism, the SMA was now the dominant arterial supply feeding blood into the hepatic artery, left gastric artery, and splenic artery via the GDA. This made the risks of the prophylactic angioembolization of the GDA outweigh the benefits, especially since there was no active bleeding noted from the IR angiogram. Subsequently, for several inpatient days, the patient did not experience any overt rebleeding episodes. However, his hemoglobin levels continued to downtrend, with the most recent at the time being 6.5 mg/dL. The patient then experienced an episode of melena, and two units of PRBC and one unit of FFP were given. Because the patient was not a candidate for angioembolization due to retrograde arterial flow secondary to MALS, it was decided that the patient would be best served with exploratory laparotomy, MAL release, and surgical ligation of the bleeding duodenal artery.

At the time of the surgical intervention, the patient’s hemoglobin level was 6.2 mg/dL. A longitudinal midline incision was made to gain access into the abdominal cavity. The common hepatic artery was identified and traced back to the celiac trunk, where the MAL was then completely incised. A transpyloric duodenotomy incision was made 4-5 cm in length. The bleeding vessel at the base of the duodenal ulcer was identified, which was actively squirting blood at the duodenal bulb and was surgically ligated. The duodenotomy was then closed in a horizontal and running fashion with an omental patch repair of the pyloroplasty. A Jackson-Pratt (JP) drain was then placed in the RUQ.

The gallbladder was incidentally found to be full of large stones, and the decision was made to perform a cholecystectomy. Pathologic evaluation of the gallbladder sample revealed chronic cholecystitis with cholelithiasis. The patient stayed in the hospital for several days with his heart rate, blood pressure, and hemoglobin levels remaining stable. An upper GI fluoroscopic study performed on postoperative day four showed irregularity of the proximal duodenum, likely secondary to postoperative edema. However, no stricture, obstruction, or gastrografin leakage was observed. The patient was no longer experiencing nausea or vomiting, was able to pass flatus, and was having normal and regular bowel movements. He was discharged on a full liquid diet, quadruple therapy, and with the RUQ JP drain in place. The patient has not returned for additional treatment or intervention.

## Discussion

In order for distal organ systems to stay perfused in the setting of MALS, a compensatory mechanism that can manifest is the formation of collateral circulation, most commonly from the existing pancreaticoduodenal anastomotic network [22]. Retrograde arterial flow through these pancreaticoduodenal arcades may result in sufficient perfusion to the distal celiac organs in periods of rest or satiety. However, “arterial steal” can develop during exercise or postprandial periods, when blood is diverted to exerting muscles or the intestines, respectfully [23]. In the setting of retrograde arterial flow from the SMA secondary to MALS, the spleen, stomach, liver, and pancreas depend on this collateral circulation. Therefore, it is important to keep the GDA and pancreaticoduodenal arcades patent and intact until definitive therapy can be taken to resolve the underlying MALS and achieve normal anterograde celiac arterial flow [24].

As pancreaticoduodenal collateral circulation is established, the increased blood flow rate can impart stress on the arterial intimal layer, in turn weakening and dilating the arterial wall to form a true aneurysm [25]. This can be a concerning development as these aneurysms are prone to rupture, which can cause life-threatening hemodynamic instability [26]. Although these secondary aneurysms are most commonly seen in the pancreaticoduodenal arcades, they can also be present in the common hepatic artery, splenic artery, dorsal pancreatic artery, jejunal artery, left gastric artery, and other vessels where there exists turbulent or abnormal blood flow [27]. Despite these aneurysms being secondary to MALS, there does not appear to be a significant association between the severity of celiac stenosis from MALS and the formation of collateral circulation or splanchnic aneurysms [27].

If splanchnic aneurysms are seen on radiographic imaging, certain interventions can be taken as prophylactic measures to prevent future rupture. Surgical MAL release can be performed as a prophylactic measure to re-establish celiac anterograde flow and relieve stress on the existing aneurysm [28]. Alternatively, the aneurysm can be treated with coil embolization in order to ensure that rupture does not occur in the future [29]. Even in the case of aneurysm rupture, MAL release can prove to be beneficial to revascularize the celiac trunk and prevent the formation of future de novo aneurysms [30]. However, it has also been documented that coil embolization of a ruptured aneurysm without additional celiac revascularization or MAL release can be an alternative option as long as there is close follow-up and serial imaging [31].

Although retrograde arterial flow through the pancreaticoduodenal arcades was observed in the presented case, aneurysms were not seen on diagnostic or interventional angiography. This provides further support for the previously mentioned study concluding that the degree of celiac stenosis may not be correlated with the likelihood of developing retrograde flow or splanchnic aneurysmal development [27]. Given the complicated clinical picture, it is unclear to what degree, if any, the patient’s MALS contributed to his acute and chronic symptoms, especially since MALS was seen almost two years prior to this encounter without manifestation of GI symptoms. However, given the patient’s severe hemorrhagic peptic ulcer disease of the duodenum, it may be the case that increased blood flow through the pancreaticoduodenal arcades could have contributed to recurrent and prolonged intestinal bleeding. Hence, it was important to achieve hemostasis of the bleeding duodenal ulcer without removing the collateral blood circulation to the distal celiac organs. Ultimately, open MAL release coupled with surgical ligation of the bleeding duodenal artery was successful at achieving hemostasis and restoring celiac arterial flow.

## Conclusions

Median arcuate ligament syndrome is a rare condition in which the celiac trunk is compressed by the median arcuate ligament, oftentimes presenting without symptoms. In the setting of both symptomatic and asymptomatic MALS, careful attention should be given to recognizing if retrograde arterial flow from the pancreaticoduodenal arcades is present. Retrograde arterial flow that sufficiently perfuses the distal celiac organs can mask symptom manifestation from MALS yet still present as a contraindication to GDA angioembolization unless celiac arterial flow is concurrently restored through other interventional means. In summary, retrograde arterial blood flow from the pancreaticoduodenal arcades observed in the setting of MALS should be recognized as a contraindication to GDA angioembolization.
